# Exploring Gadolinium-Based Contrast Media for Retrograde Pyelography in the Context of Iodine Allergy

**DOI:** 10.7759/cureus.47439

**Published:** 2023-10-21

**Authors:** Firaas A Khan, Joseph Balbona, David J Hernandez

**Affiliations:** 1 Morsani College of Medicine, University of South Florida, Tampa, USA; 2 Department of Urology, University of South Florida, Tampa, USA

**Keywords:** gadolinium-based contrast agent, ceus retrograde pyelography, kidney stone disease, renal stone disease, minimal invasive and endourology

## Abstract

Iodinated contrast media can rarely cause severe allergic reactions during nonvascular urologic imaging procedures. Alternatives like gadolinium-based contrast may help mitigate this risk in susceptible patients. A 66-year-old woman with a documented iodine allergy presented with an obstructing stone in the right ureter. To avoid the risk of an allergic reaction, the decision was made to use an alternative non-iodinated contrast agent for retrograde pyelography prior to ureteral stent placement. Gadobenate dimeglumine, an MRI contrast agent, was diluted 50:50 with saline and utilized successfully to provide adequate opacification for safe stent placement without adverse reaction. The patient underwent repeat pyelography with gadobenate dimeglumine one month later during ureteroscopy without complication. This case demonstrates that diluted gadobenate can serve as an effective alternative to iodinated contrast media in patients at high risk of reaction to iodine-containing agents. While severe reactions to iodinated contrast are uncommon in nonvascular urologic procedures, they can still occur even with premedication. Gadolinium-based agents have been reported to provide sufficient opacification for most urologic interventions, though inferior radiographically to iodinated contrast. Further study on gadolinium efficacy and safety in this setting is warranted. However the present case supports gadobenate dimeglumine as a viable option for retrograde pyelography when allergy risk precludes iodinated contrast use.

## Introduction

Allergies to iodinated contrast media (ICM) pose a challenge in clinical practice as the risk of allergen exposure needs to be weighed against the benefit of contrast-enhanced imaging. The incidence of adverse reactions to ICMs in nonvascular diagnostic procedures like retrograde pyelography is low and has been recorded to be between 0.26% [1] and 2.8% [2]. An iodine allergy is often reported by patients or previously documented in medical records, though the validity and severity of their allergy may be unclear. Patients may erroneously report an ICM allergy due to a shellfish allergy, the association of which is now considered unfounded [1]. Notwithstanding the reduced risk in nonvascular procedures as well as the limited reliability of a reported or documented allergy to ICM, observed adverse reactions to nonvascular ICM use can range from mild reactions to severe anaphylaxis. As the latter can be life-threatening, there remains a diagnostic dilemma even in non-vascular ICM use to avoid anaphylaxis. Ultimately, the decision to utilize ICM lies with the physician. While not routinely used in Urologic imaging, non-iodinated contrast media can serve as an alternative to ICM in patients at the highest risk for a severe adverse reaction to traditional agents.

## Case presentation

**Figure 1:**
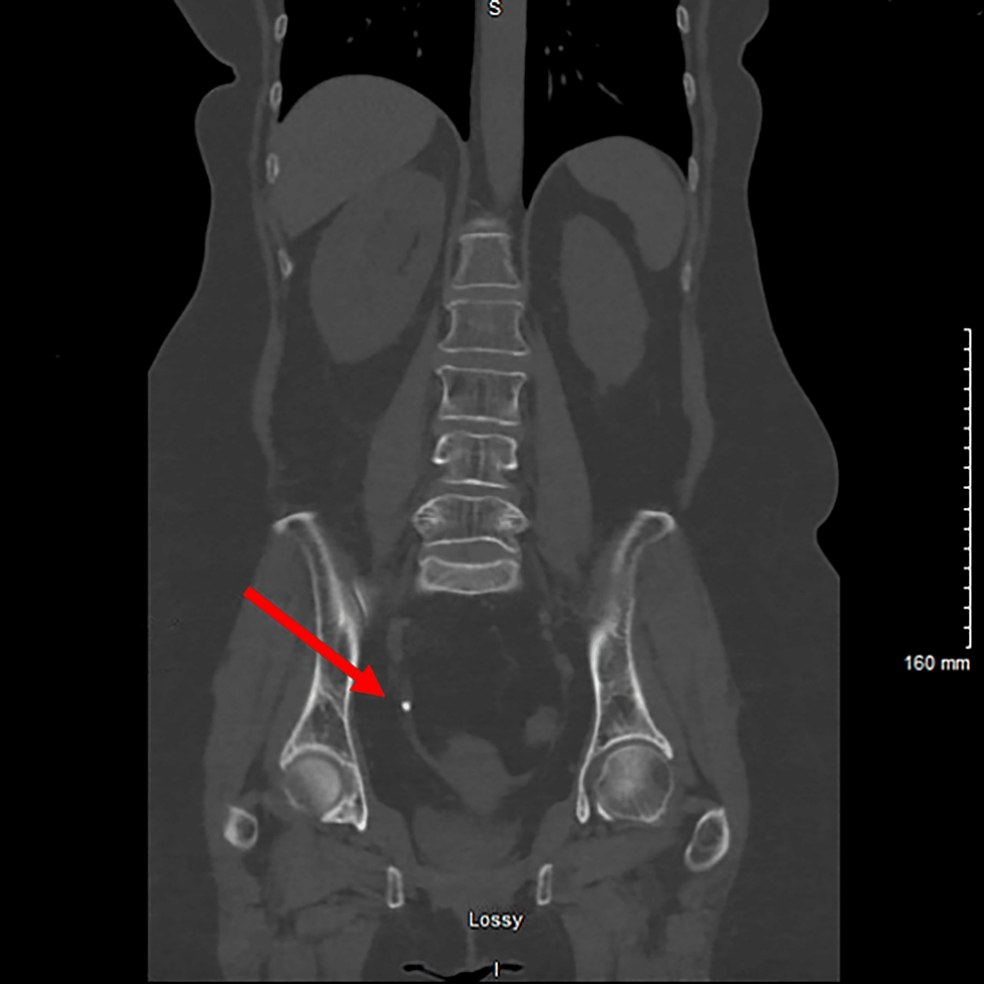
Coronal View - Obstructing 5mm Stone in Distal Right Ureter and Hydronephrosis The arrow indicates the 5mm obstructing stone in the distal right ureter.

**Figure 2:**
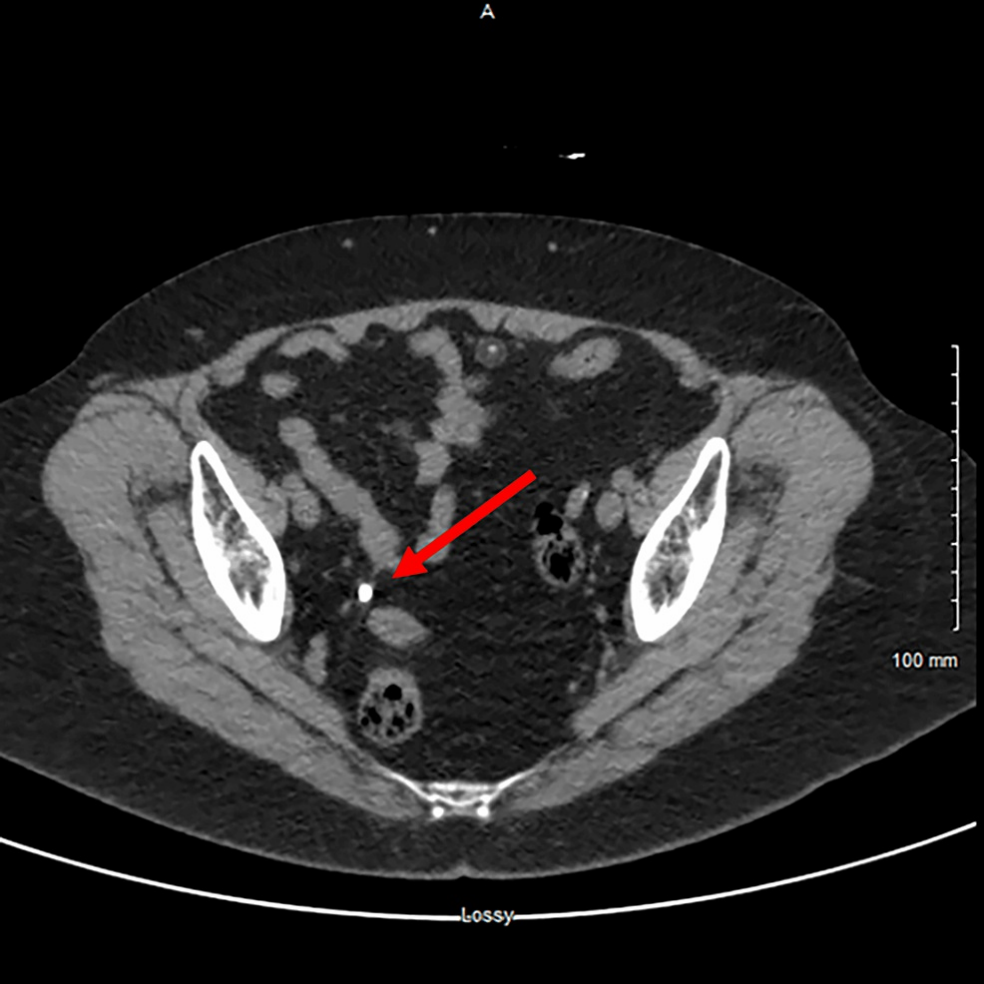
Transverse View - Obstructing 5mm Stone in Distal Right Ureter The arrow indicates the 5mm obstructing stone in the distal right ureter.

A 66-year-old female with a documented iodine allergy and no significant past medical history presented to the Emergency Department with right flank pain for one-week duration. While she had no associated fever, she had previously been treated with three courses of antibiotics for a suspected urinary tract infection (UTI) by urgent care providers. Labs showed creatinine 1.2 mg/dL, glomerular filtration rate (GFR) 50 mL/min, white blood cells (WBCs) 8.18 thou/cmm, and negative urine cultures. Urinalysis was positive for leukocyte esterase and WBCs. A computed tomography (CT) scan of the patient's abdomen and pelvis without contrast was obtained which revealed a 5mm obstructing stone in the distal right ureter with associated perinephric and periureteral stranding and moderate right hydroureteronephrosis. The obstructing stone in the distal right ureter was visualized from different angles in the coronal view (Figure 1) and transverse view (Figure 2).

**Figure 3:**
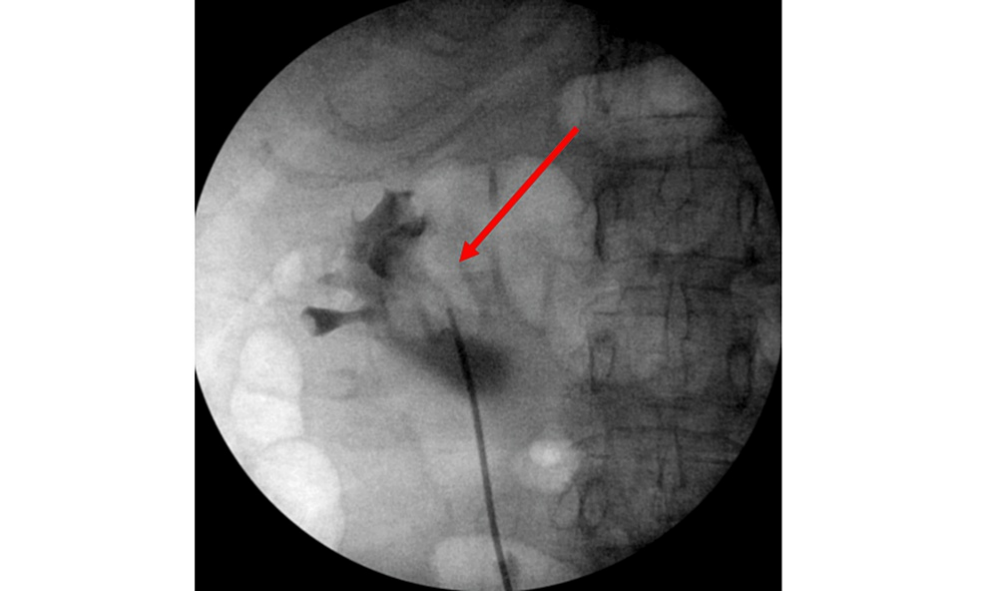
Representative Retrograde Pyelogram The arrow highlights the opacified renal collecting system, enhancing visibility of internal kidney structures, including the renal pelvis and major calyces using a gadolinium-based contrast agent.

Given her positive urinalysis (UA) and consistent pain, she elected to pursue endoscopic intervention with ureteral stent placement. The patient was admitted for pain control and underwent stent placement the following day. As she had a reported history of anaphylaxis to iodine and had not recently received intravenous iodinated contrast, the decision was made to perform retrograde pyelography with an alternative agent. Gadobenate-dimegluine MRI contrast (529 mg/ml) was utilized. As the contrast showed excellent radiopacity on a test image of the contrast medium (not shown), the medium was diluted in a 50/50 ratio with normal saline for procedural use. A representative retrograde pyelographic image is displayed in Figure 3.

Adequate opacification of the collecting system was noted, demonstrating moderate hydroureteronephrosis from the obstructing distal ureteral stone. Using the retrograde imaging, a 6x28 JJ stent was placed without difficulty. No adverse events were encountered during the procedure. One month later, the patient underwent stent removal, ureteroscopy with laser lithotripsy, and stone removal in which retrograde imaging was also performed using gadobenate-dimegluine in the same ratio. At her two-month follow-up appointment, the patient reported complete resolution of her presenting symptoms.

## Discussion

Severe reactions to ICMs are rare. In 76,174 patients who had undergone non-intravenous urinary tract imaging, 0.48% developed adverse contrast reactions. Of those patients, only 13% had severe anaphylactoid reactions [3]. To limit adverse reactions to ICMs during nonvascular urologic diagnostic imaging, prophylactic steroid or antihistamine treatment is often initiated before administering these iodinated agents in patients with a reported allergic reaction to contrast. In a survey of practicing urologists, 24.8% and 23.4% of respondents stated they would administer an antihistamine or steroid as pretreatment, respectively for patients with a known IV contrast allergy before proceeding with studies such as retrograde pyelogram [2]. Efficacy to prophylactic treatment has been demonstrated in several studies, such as Lasser et al. (1994) [4] which showed a reduction in the incidence of reactions from 4.9 to 1.7% when 32 mg of oral methylprednisolone was given six to 24 hours before contrast administration and again two hours before ICM administration. Despite improvement with pre-medication, patients can still suffer severe reactions following non-vascular contrast administration. Although most literature shows that the incidence of adverse reactions to ICMs in intraluminal studies appears to be extremely low, such as in Joseph et al. (2021) [5] that found no evidence of allergic reaction to ICMs administered in 86 patients for endourologic procedures with a known history of adverse events to ICMs, urologists need to be aware that reactions may still develop. Many physicians are aware of the risk and are hesitant to administer ICMs to patients with a known shellfish allergy or a history of adverse reactions to contrast. In Dai et al. (2018) [2], 8.3% of respondents to a survey confirmed they would not administer ICM at all in patients with a known past allergic reaction. This illustrates the need to devise non-iodine-containing contrast alternatives for patients with a known history of adverse reactions to ICMs. This issue may be even more critical in outpatient surgery centers providing endourologic procedures as management of anaphylaxis may be more tenuous as compared to hospital-based practice.

In patients at risk of adverse reactions from ICMs, the use of gadolinium-based contrast media (GBCM) should be considered as a possible alternative. In Spinosa et al. (2000) [6], three patients with a history of allergic reactions from ICMs underwent various uroradiologic procedures including a diagnostic nephrostogram, antegrade pyelography with the placement of a nephroureteral stent, and percutaneous access for nephrostolithotomy. Researchers found that the degree of opacification in the intrarenal renal collecting system and ureter was inferior to that with ICMs however, it was sufficient to allow these interventions to be performed safely. In another study, gadopentetate dimeglumine was used in an X-ray urethrography to sufficiently exclude a suspected urethral diverticulum [7].

Currently, GBCM has been deemed most suitable in MRI imaging because gadolinium is a heavy metal that forms a 3+ charge with seven unpaired electrons that serve to attract nearby protons. This complexation reduces the relaxation time of nearby protons in a magnetic field at low concentrations to provide enhanced image quality important for tissue differentiation in disease diagnosis [8]. In X-ray applications, gadolinium has attenuation properties that are similar to that of iodine-53 during CT [9].

GBCMs are approximately five to 10 times more expensive per milliliter than ICMs. However, in patients at risk for a serious reaction to ICM or contrast-induced nephropathy, the added costs of the gadol may outweigh the additional hospital costs related to a prolonged hospital stay for severe contrast reaction or contrast-induced nephropathy. For those with experience using carbon dioxide as an alternative contrast agent, this agent can also be used to identify the intrarenal collecting system but can be difficult to visualize and frequently produces discomfort at the site of injection. Injection of room air into the kidney is not advisable unless injected directly into a closed intrarenal collecting system since inadvertent administration into the vessels could result in an air embolus.

In conclusion, GBCMs can be used as an alternative contrast material during interventional uroradiologic procedures in patients with a history of a severe contrast reaction to ICM, or in patients in whom there is a concern that administration of ICM may lead to adverse events. Drawbacks to the use of GBCMs include their lower attenuation compared to iodine-based agents. With lower attenuation, GBCM is less able to be diluted compared to ICM [10]. However, as demonstrated in this report, the luminal opacification generated by GBCMs is generally sufficient for retrograde pyelography.

Of note, ultrasound-guided stent placement is a common alternative for patients with contraindications to ICMs; however, it has inherent limitations. These include limited visualization of deep structures, wires, or areas obstructed by bone or gas, lack of contrast enhancement, and reliance on operator skill in deploying stents effectively [11]. In cases where ICM is contraindicated, GBCM may offer a more viable and effective alternative for precise opacification and guiding ureteral stent placement safely.

In this report, the agent gadobenate-dimeglumine (Gd-BOPTA or Multihance) was utilized. Other GBCMs have been shown to be efficacious in urologic diagnostic studies including retrograde using formulations like gadoteridol [12] and gadobutrol (Gadovist) [13]. In our study we utilized Multihance diluted in a 50/50 ratio with saline as opacification of the collecting system was required only to guide ureteral stent placement. This is an off-label use of the product that is not approved by the U.S. FDA. When more detailed images are required, such as in diagnostic retrogrades to rule out upper tract malignancy, undiluted contrast may be considered for better opacification of the collecting system.

The risks associated with intravascular GBCM use include nephrogenic systemic fibrosis and gadolinium deposition disease [8]. These complications have not been described with non-intravenous use. This may be secondary to the rarity of these complications and the infrequency of non-vascular utilization of GBCMs. This may be alternatively secondary to a lower systemic absorption through the urinary tract, although this has not been studied. Hypersensitivity reactions associated with GBCM administration are uncommon and occur in 0.004%-0.7% of cases [14]. The most frequent reactions are immediate reactions where skin manifestations are presented in 75-100% of cases, including urticaria, rashes, pruritus, and limited facial edema [15]. Anaphylaxis occurs in 0.01% of IV injections but has not been reported for non-vascular examinations [16].

## Conclusions

Gadobenate-dimeglumine is an effective contrast agent to visualize the urinary collecting system in cases where ICMs are contraindicated due to a patient’s prior history of adverse reactions.
